# The effect of repetitive transcranial magnetic stimulation on the Hamilton Depression Rating Scale-17 criterion in patients with major depressive disorder without psychotic features: a systematic review and meta-analysis of intervention studies

**DOI:** 10.1186/s40359-024-01981-6

**Published:** 2024-09-10

**Authors:** Elham Hassanzadeh, Ghobad Moradi, Modabber Arasteh, Yousef Moradi

**Affiliations:** 1 Research Committee, Kurdistan University of Medical Sciences, Sanandaj, Iran; 2 Social Determinants of the Health Research Center, Research Institute for Health Development, Kurdistan University of Medical Sciences, Sanandaj, Iran; 3 Department of Psychiatry, School of Medicine, Kurdistan University of Medical Sciences, Sanandaj, Iran

**Keywords:** Repetitive transcranial magnetic stimulation, Hamilton depression rating scale-17, Major depressive disorder, Evidence synthesis

## Abstract

**Aim:**

In line with the publication of clinical information related to the therapeutic process of repetitive transcranial magnetic stimulation (rTMS) and the updating of relevant treatment guidelines, the present meta-analysis study was designed and conducted to determine the effect of repetitive transcranial magnetic stimulation (rTMS) on the Hamilton Depression Rating Scale-17 (HDRS-17) criterion in patients with major depressive disorder (MDD) without psychotic features.

**Methods:**

In this study, a systematic search was conducted in electronic databases such as PubMed [Medline], Scopus, Web of Science, Embase, Ovid, Cochrane Library, and ClinicalTrials. gov using relevant keywords. The search period in this study was from January 2000 to January 2022, which was updated until May 2023. Randomized controlled trials (RCTs) that determined the effect of repetitive transcranial magnetic stimulation (rTMS) on the Hamilton Depression Rating Scale-17 (HDRS-17) criterion in patients with major depressive disorder (MDD) without psychotic features were included in the analysis. The quality of the included RCTs was assessed using the Cochrane Risk of Bias checklist. Statistical analyses were performed using STATA (Version 16) and RevMan (Version 5).

**Results:**

Following the combination of results from 16 clinical trial studies in the present meta-analysis, it was found that the mean Hamilton Depression Rating Scale-17 (HDRS-17) in patients with major depressive disorder (MDD) decreases by an average of 1.46 units (SMD: -1.46; % 95 CI: -1.65, -1.27, I _square_: 45.74%; P _heterogeneity_: 0.56). Subgroup analysis results indicated that the standardized mean difference of Hamilton Depression Rating Scale-17 (HDRS-17) varied based on the number of treatment sessions: patients receiving 10 or fewer repetitive transcranial magnetic stimulation (rTMS) sessions showed a mean Hamilton Depression Rating Scale-17 (HDRS-17) reduction of 2.60 units (SMD: -2.60; % 95 CI: -2.86, -2.33, I _square_: 55.12%; P _heterogeneity_: 0.55), while those receiving 11 to 20 sessions showed a mean Hamilton Depression Rating Scale-17 (HDRS-17) reduction of 0.28 units (SMD: -0.28; % 95 CI: -0.65, -0.09, I _square_: 39.91%; P _heterogeneity_: 0.89).

**Conclusion:**

In conclusion, our meta-analysis demonstrates the efficacy of repetitive transcranial magnetic stimulation (rTMS) in reducing depressive symptoms in major depressive disorder (MDD) patients. The complex results of subgroup analysis revealed insight on the possible benefits of a more focused strategy with fewer sessions, as well as the impact of treatment session frequency. These findings add to our understanding of repetitive transcranial magnetic stimulation (rTMS) as a therapeutic intervention for the treatment of major depressive illnesses.

**Supplementary Information:**

The online version contains supplementary material available at 10.1186/s40359-024-01981-6.

## Introduction

Depression is a common and growing mental disorder that affected more than 264 million people worldwide in 2020 [1, 2]. Depression is classified as a mood disorder and encompasses a wide range of symptoms, from fatigue and loss of energy to decreased interest, significant weight loss or gain, changes in sleep patterns, and even suicidal thoughts. It affects the individual’s cognitive, emotional, physical, and social aspects of life. Depression is a common and growing mental disorder that will affect over 264 million people worldwide in 2020. Depression is classified as a mood disorder and encompasses a wide range of symptoms, from fatigue and decreased energy to decreased interest, significant changes in weight and sleep patterns, and suicidal thoughts, which affect the individual’s cognitive, emotional, physical, and social life [3–6]. Previous studies have shown that the global prevalence of major depressive disorder (MDD) increased by approximately 13% from 2007 to 2017 [7]. The World Health Organization (WHO) ranks MDD as the 11th leading cause of disability and death in the world [8]. Although patients may have relatively better occupational and social functioning between episodes, the risk of relapse is high. Without treatment, a depressive episode lasts about 6 months to 1 year, and with appropriate treatment, it can be reduced to about three months. Currently, the treatment of depression primarily involves three methods: medication therapy, psychotherapy, and electroconvulsive therapy. Antidepressant medications play a major role in the treatment of depression. The most commonly used drugs in depression treatment include the drug classes SSRI (selective serotonin reuptake inhibitor), TCA (tricyclic antidepressant), and SNRI (selective serotonin-norepinephrine reuptake inhibitor). To choose the appropriate antidepressant drug, drug side effects, its compatibility with the patient’s age, physical condition, and target symptoms should be considered. For example, in elderly depressed individuals or those with heart disease, due to anticholinergic side effects of drugs and changes in blood pressure status, the use of SSRI and SNRI is preferred over TCA because they have fewer side effects. In psychotherapy, various methods, such as cognitive therapy, interpersonal psychotherapy, and behavioral therapy, are used to treat depression. These methods are usually much more effective in combination with medication therapy [9–12]. Currently, depression is primarily treated through three methods: medication therapy, psychotherapy, and electroconvulsive therapy. Antidepressant medications play a crucial role in the treatment of depression. Many patients with MDD do not respond to standard treatment with medication therapy and psychotherapy [13, 14]. Neuromodulation techniques, including non-invasive methods such as repetitive transcranial magnetic stimulation (rTMS) and invasive methods such as electroconvulsive therapy (ECT), are being considered as potential treatments for depression [15, 16]. ECT is a method that uses a low electrical current to induce a generalized cerebral seizure under general anesthesia. This method is mainly used for the treatment of severe depression, but it is also used for other conditions such as bipolar disorder, schizophrenia, schizoaffective disorder, catatonia, and malignant neuroleptic syndrome [17]. rTMS is a non-invasive neuromodulatory technique that is used in the treatment of a wide range of neurological disorders, including depression. This technique involves the application of a magnetic field to specific areas of the brain outside the skull in order to modify neural excitability [18]. In rTMS, magnetic pulses with different shapes and frequencies (usually between 1 and 20 Hz) are delivered to specific regions of the brain to determine changes in excitability. The standard rTMS method typically involves high-frequency (HF) stimulation of the left dorsolateral prefrontal cortex (DLPFC) of the frontal lobe. However, this standard method is not beneficial for all patients with depression [7]. Evidence shows that the response rate in patients who receive standard treatment is, on average, 29.3% [19, 20]. Therefore, new forms of rTMS are necessary to increase the rate of improvement in patients with depression, especially treatment-resistant depression [19, 20]. rTMS is typically used when standard treatments such as medications and psychotherapy are not effective. This method does not require surgery or electrode implantation, and unlike electroconvulsive therapy (ECT), which is routinely used for depression, it does not cause seizures and does not require anesthesia. Generally, rTMS is considered a safe and well-tolerated method. However, it can cause some side effects. The side effects are usually mild to moderate and improve shortly after a session and decrease over time with additional sessions. Common side effects of this method include headaches, discomfort at the site of stimulation on the scalp, tingling, muscle spasms or contractions of facial muscles, and light-headedness [21–23]. Recently, the use of rTMS for MDD and treatment-resistant depression has gained considerable attention. Although rTMS is considered a promising therapeutic option for MDD, the clinical response to it is partial, indicating the need for a more comprehensive understanding of the pathophysiology of MDD and the mechanisms involved in the therapeutic process of rTMS. Therefore, in order to disseminate clinical information regarding the therapeutic process of rTMS and update related treatment guidelines, the present meta-analysis was designed and conducted to determine the effect of rTMS on assessment measures of non-psychotic MDD.

## Methods

The present study was a systematic review and meta-analysis conducted according to the Preferred Reporting Items for Systematic Reviews and Meta-Analyses (PRISMA) guidelines [24].

### Search strategy and selection process

At the beginning of the study, relevant keywords were determined based on the study’s title and objectives, and a search strategy was developed and designed for each database by combining these keywords using the AND/OR operators. The primary keywords included “repetitive transcranial magnetic stimulation”, “Hamilton Depression Rating Scale”, “major depression disorder”, and related synonyms were found using Thesaurus, Emtree, and Mesh. In this study, the following databases were searched: PubMed [Medline], Scopus, Web of Science, Embase, Ovid, Cochrane Library, and ClinicalTrials. gov. The search period in this study was from January 2000 to January 2022, which was updated until May 2023. In addition to the electronic databases, a manual search was conducted using reference checking and selected related studies to ensure that no relevant studies were missed. After completing the search, all retrieved studies were imported into Endnote software version 8, and screening of studies was performed based on title, abstract, and full text. The search strategy process was conducted by two independent reviewers (EH and GH), and any discrepancies were resolved through discussion or consultation with a third reviewer (YM).

### Eligibility criteria

The inclusion criteria for this study were determined based on the PICOT structure. Therefore, studies included in this systematic review and meta-analysis were those that included populations with MDD, used rTMS as the main intervention, compared it with placebo (sham stimulation), and had outcomes that included improvement in the HDRS assessment of MDD (Table 1). The studies considered were randomized controlled trials with an intervention design. The exclusion criteria included duplicate citations, review articles, cross-sectional studies, case-control or cohort studies, books, conference papers, and clinical trials with different primary outcomes and interventions.

**Table 1:** The criteria for inclusion of studies in the present meta-analysis

Type of study (T)	Outcomes (O)	Comparison (C)	Intervention (I)	Population (*P*)
All clinical trial studies	The mean of total bilirubin and phototherapy length	The comparison group included other drugs or placebo.	The desired intervention in the present meta-analysis was albumin administration before pre-exchange plasma	The target population in this meta-analysis was neonatal with hyperbilirubinemia.

### Data extraction process

Finally, data extraction was performed considering items related to the studies (authors’ names, year of publication, study design, sample size, and country of study), items related to the target population (type of depression in the patients under study), items related to the intervention (number of sessions, frequency used), the comparison group, and the outcomes of interest (level of improvement in HDRS score). The selection and data extraction process was conducted by two independent reviewers (EH and GH/MA), and any discrepancies were resolved through discussion or consultation with a third reviewer (YM).

### Studies risk of bias

After screening, the selected studies were assessed for quality or risk of bias using Version 2 of the Cochrane risk-of-bias tool for randomized trials (RoB 2) [25]. The RoB 2 tool evaluates the risk of bias in five domains, including randomization process, deviations from intended interventions, missing outcome data, measurement of the outcome, and selection of the reported result. For each domain, the study was rated as low, high, or unclear risk of bias. By using the RoB 2 tool, the authors were able to assess the quality of the selected studies and ensure that the study findings were reliable and accurate. The selection and data extraction process was conducted by two independent reviewers (YM and GH).

### Synthesis method

In this study, STATA software version 17 was used to conduct the meta-analysis. The desired index for the analysis was the standardized mean difference (SMD). To calculate this index, the mean and standard deviation (SD) before and after the intervention in each group of the selected studies were extracted, and their difference was calculated. Then, using the fixed-effect model (FEM) in STATA software, this index was calculated. Additionally, for outcomes in which the baseline mean was not reported in both intervention and comparison groups, the weighted mean difference (WMD) index was used. In this index, the mean and SD of the outcome in the intervention and comparison groups were compared, and their difference was calculated by considering the weight of each study. To evaluate publication bias, the Egger’s test was used, and to assess heterogeneity, the I-square and Q Cochrane test were used. Subgroup analyses were also performed based on the number of treatment sessions, device power, and study population. A significance level of less than 0.05 was considered in this meta-analysis. In addition, the Revman software version 5 was also used to assess the risk of bias and draw related figures.

## Results

**Fig. 1:**
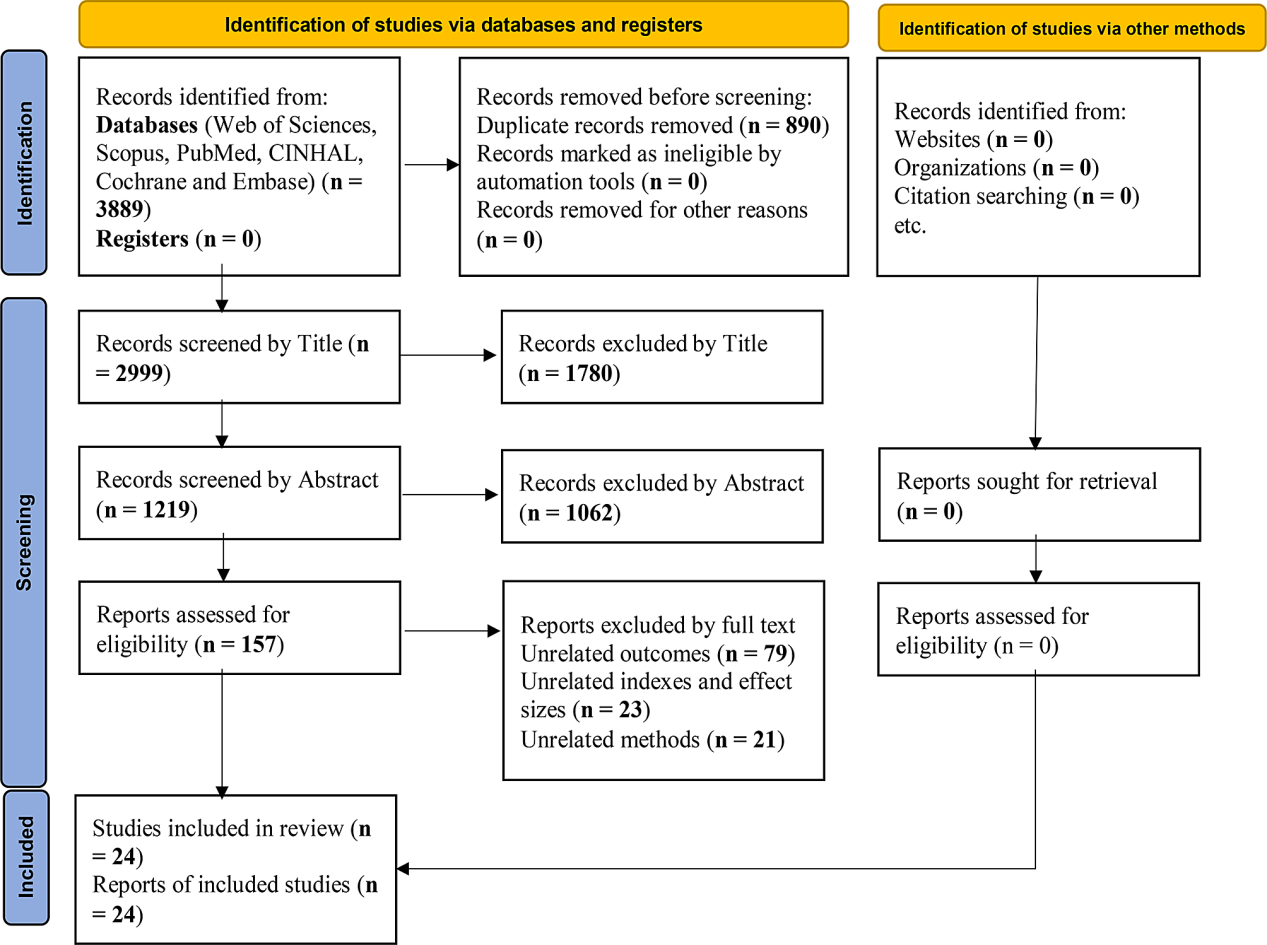
A flow diagram demonstrating the study selection process based on PRISMA 2020 flow diagram

At the beginning of the study, a total of 3,889 articles were retrieved through the search process. In the screening stage, based on the title and abstract, 1,780 and 1,062 articles were excluded, respectively. In the full-text screening stage, 157 articles were reviewed, of which 133 were excluded due to unrelated outcome (79 articles), unrelated effect size (23 articles), and unrelated methodology (21 articles). Finally, 24 relevant clinical trials remained for analysis [26–50] (Fig. 1). Clinical and basic information, the target population, and other relevant information for the selected studies are reported in Table 2. Among the selected studies, four clinical trials did not report the SMD index (Table 2).

**Table 2:** The characteristics of included studies

Authors (*R*)	Years (Type of studies)	Sample size			Intervention (Tool, Tesla)	Comparison (Drug, and…)	Study Population	HDRS (HAMD)		MARDs		BDI		FU	
		T	I	*P*											
								I (%)	*P* (%)	I (%)	*P* (%)	I (%)	*P* (%)		
1- Y.C. Tsai, et al. [1].	2021	42	20	22	10 Hz rTMS (1,600 pulses/session − 10 sessions)	sham stimulation 10 sessions	TRD patients	BL: 22.60 ± 3.33	BL:22.59 ± 2.61	NR	NR	NR	NR	2 weeks	
								Change:30.19 ± 6.26	Change:14.75 ± 2.94						
2- Y.C. Tsai, et al. [1].	2021	41	19	22	prolonged iTBS (1,800 pulses/session − 10 sessions)	sham stimulation 10 sessions	TRD patients	BL:22.53 ± 3.17	BL:22.59 ± 2.61	NR	NR	NR	NR	2 weeks	
								Change:40.85 ± 6.70	Change:14.75 ± 2.94						
3- C. Plewnia, et al. [2].	2021	236	118	118	iTBS (600 pulses/190s) combined with cTBS (600 pulses/40s) 30 sessions	sham stimulation 30 sessions	MDD Patients	NR	NR	NR	NR	NR	NR	6 weeks	
5- M. Majdi, et al. [3].	2021	30	15	15	rTMS (10 Hz, 20 sessions)	and those in the control group were put on a waiting list for training	MDD Patients	NR	NR	NRE	NRE	BT: 29.3			
												AT: 19.4			
7- A. Holczer, et al. [4].	2021	20	10	10	cTBS 600 pulses + iTBS 600 pulses (10 sessions)	Sham stimulation (10 sessions)	Unipolar MDD	BL:19.5 ± 5.7	BL: 15.0 ± 4.3	NR	NR			One day	
								?	?						
9- P. E. Croarkin, et al. [5].	2021	103	48	55	TMS (10 Hz, 30 sessions)	Sham stimulation (30 sessions)	TRD	BL:28.8 ± 5.75	BL:29.5 ± 6.69	NR	NR			6 weeks	
								Change:18.1 ± 10.91	Change:19.2 ± 11.03						
11- P. F. P. van Eijndhoven, et al. [6].	2020	31	15	16	rTMS (10 Hz, 20 sessions)	Sham rTMS (20 sessions)	Chronic TRD	BL: 24.1 ± 4.2	BL: 22.7 ± 3.8	NR	NR			1 weeks	
								AT: 21.0 ± 5.4	AT: 18.6 ± 4.2						
13- J. Persson, et al. [7].	2020	23	11	12	Active iTBS (20 sessions)	Sham iTBS (20 sessions)	Unipolar depression and bipolar depression			BL: 29.7 ± 6.2	BL: 28 ± 8.4			4 weeks	
										Change: 6.3 ± 9.1	Change: 3.8 ± 6.2				
14- J. H. Hwang, et al. [8].	2020	13	6	7	rTMS (10 Hz, 3 times a week, for 4 weeks)	Sham (1-wing 90-degree method)	depressed hemodialysis patients			NR	NR	BL: 21.2 ± 7.4	BL: 24.0 ± 10.7	4 weeks	
												AT: 13.8 ± 5.1	AT: 18.4 ± 9.9		
15- L. L. Dai, et al. [9].	2020	103	48	55	Active rTMS (10 Hz, 5 times per week for 4 weeks)	Sham rTMS	Elderly depression patients	Effective rate: 25/48, 52.1%	Effective rate: 18/55, 32.7%	NR	NR			2 weeks	
								Effective rate: 45/48, 93.8%	Effective rate: 46/55, 83.6%	NR	NR			4 weeks	
16- S. H. Siddiqi, et al. [10].	2019	14	9	5	Active bilateral rTMS (20 sessions, left side: 4000 pulses/ 10 Hz, right side: 1000 pulses, 1 Hz)	sham treatment with a Magstim Rapid-2 stimulator	Patients with MDD secondary to TBI			NR	NR				
17- V. Rao, et al. [11].	2019	30	13	17	LFR rTMS (20 sessions, 1,200 pulses/session, 1 Hz)	sham treatment	TBI depression Patients	BL: 23.2 ± 4.4	BL: 23.7 ± 4.4	NR	NR			16 weeks	
								AT:	AT:						
18- S. N. Light, et al. [12].	2019	19	8	11	Active rTMS (20 sessions, 3000 pulses, 10 Hz)	Sham treatment (20 sessions, 3000 pulses, 10 Hz)	MDD	NR	NR	BL: 23.75 ± 5.06	BL: 21.72 ± 3.87			4 weeks	
19- S. Lee, et al. [13].	2019	30	16	14	rTMS (15 sessions, 3000 pulses, 10 Hz)	Sham rTMS (15 sessions)	Unipolar MDD	BL: 21.44 ± 5.21	BL: 19.02 ± 6.40	NR	NR	BL:28.5	BL:27.79		
								Change:	Change:			Change:	Change:		
20- F. Leblhuber, et al. [14].	2019	29	19	10	Active rTMS (10 sessions, 30 min, 3 Hz)	Sham rTMS (10 sessions, 30 min, 3 Hz)	TRD	BL: 12.9 ± 0.89	BL: 13.2 ± 1.43	NR	NR				
								AT: 10.2 ± 0.67	AT: 13.3 ± 1.48						
21- D. R. Kim, et al. [15].	2019	20	11	9	Active rTMS (20 sessions, 15 min, 5 days per week, 1 Hz, 900 pulses)	Sham rTMS (20 sessions, 15 min, 5 days per week)	Pregnant MDD	BL: 23.18 ± 3.54	BL: 22.27 ± 2.65	NR	NR	NR		NR	
								AT: 9.27 ± 6.05	AT:13.18 ± 8.00						
22- K. Jang, et al. [16].	2019	35	19	16	Active rTMS (15 sessions, 30 min, 3000 pulses, 10 Hz, 3 weeks)	Sham rTMS	Unipolar MDD	BL: 21.00 ± 5.12	BL: 19.31 ± 6.10	NR	NR	NR	NR	3 weeks	
								AT: 15.47 ± 6.32	AT: 15.38 ± 6.18						
23- F. Leblhuber, et al. [17].	2021	38	21	17	Active rTMS (10 sessions, 2400 stmiuli,30 min, 20 Hz)	Sham rTMS (10 sessions, 2400 stimuli,30 min, 20 Hz)	TRD	BL: 13.6 ± 0.96	BL: 11.4 ± 1.23	NR	NR	NR	NR	NR	
								AT: 8.0 ± 1.09	AT: 11.5 ± 1.01						
24- Zh. Zhang, et al. [18].	2021	47	24	23	Individualized rTMS (10 Hz, 1600 pulse, 20 min, twice per day 5 days)	Sham rTMS	MDD	BL: 33.79 ± 6.31	BL: 35.70± 9.28	NR	NR	NR	NR	4 weeks	
								AT:	AT:						
25- Zh. Zhang, et al. [18].	2021	50	27	23	Standard rTMS	Sham rTMS	MDD	BL: 35.81 ± 7.90	BL: 35.70 ± 9.28	NR	NR	NR	NR	4 weeks	
								AT:	AT:						
27- C. Li, et al [19].	2021	70	35	35	piTBS monotherapy (50 Hz, 1800 pulses, 10 sessions)	Sham stimulation	MDD	BL: 22.5 ± 3.5	BL: 23.1 ± 3.5	NR	NR	NR	NR	2 weeks	
								W2:17.7 ± 5.8	W2:20.0 ± 5.8						
								W14: 13.5 ± 6.6	W14: 20.1 ± 5.8						
28- C. Li, et al. [19].	2021	70	35	35	r TMS monotherapy (10 Hz, 10 sessions)	Sham stimulation	MDD	BL: 22.9 ± 3.8	BL: 23.1 ± 3.5	NR	NR	NR	NR	2 weeks	
								W2: 15.2 ± 7.0	W2:20.0 ± 5.8						
								W14: 15.6 ± 7.2	W14: 20.1 ± 5.8						
29- B. Hordacre, et al. [20].	2021	11	6	5	High-frequency rTMS (10 Hz, 3000 pulses/sessions, 10 sessions)	Sham rTMS	Post stroke Depression	NR	NR	NR	NR	BL: 23.0 ± 7.9	BL		
												AT:	AT:		
30- Y. Matsuda, et al. [21].	2020	38	18	20	dTMS (18 Hz, 1980 pulses/session, 20 sessions, 4 weeks)	Sham dTMS (18 Hz, 1980 pulses/session, 20 sessions, 4 weeks)	MDD and bipolar disorder types I or II in an acute major depressive episode	BL: 19.4 ± 8.2	BL: 20.5 ± 4.1	NR	NR	NR	NR	NR	
								Change: W4: − 4.45 (–7.93 to − 0.96) W6: − 5.53 (–9.50 to − 1.55)	Change: W4: − 0.22 (–3.74 to 3.30) W6: − 0.26 (–3.75 to 4.27)						
32- K. Dunlop, et al. [22].	2020	108			Active high frequency rTMS (20 Hz, 30 sessions)	Sham rTMS	TRD	NR	NR	NR	NR	NR	NR	NR	
33- K. Dunlop, et al. [22].	2020				Active low frequency rTMS (1 Hz, 30 sessions)	Sham rTMS	TRD	NR	NR	NR	NR	NR	NR	NR	
34- P. H. Chou, et al. [23].	2020	53	27	26	Bilateral TBS monotherapy (600 cTBS stimuli to the right DLPFC + 600 iTBS stimuli to the left DLPFC, 10 sessions, 3 weeks)	Sham stimulation (10 sessions, 2 weeks)	MDD	BL: 24.3 (3.9)	BL: 24.8 (5.3)	NR	NR	NR	NR	24 weeks	
								Change: w24: −62.7 (-18.1)	Change: w24: −36.6 (-21.2)						
36- K. E. Hoy, et al. [24].	2019	18			rTMS (right DLPFC: 1 Hz, 900 pulses + left DLPFC: 10 Hz. 1500 pulses, 20 sessions, 4 weeks)	Sham stimulation (right DLPFC: 1 Hz, 900 pulses + left DLPFC: 10 Hz. 1500 pulses, 20 sessions, 4 weeks)	Post TBI depression	NR	NR	BL: 33.64	BL: 34.40			4 weeks	
										AT: 27.10	AT: 24.13				

R: References, T: Total, I: Intervention, P: Placebo, HDRS-17: Hamilton depression rating scale-17, MARDs: Montgomery asberg depression rating, BDI: Beck depression inventory, SIS: suicidal ideation scale, Dep: Depression, ES: Effect Sides, FU: follow up, rTMS: repetitive transcranial magnetic stimulation, iTBS: intermittent theta burst stimulation, cTBS: continuous theta burst stimulation, TRD: treatment resistant depression, MDD: major depressive disorder, BL: baseline, BD: bipolar depression, BT: before treatment, AT: after treatment, TBI: traumatic brain injury, LFR: low-frequency right-sided, dTMS: deep Transcranial Magnetic Stimulation, sTMS: Synchronized transcranial magnetic stimulation

**Fig. 2:**
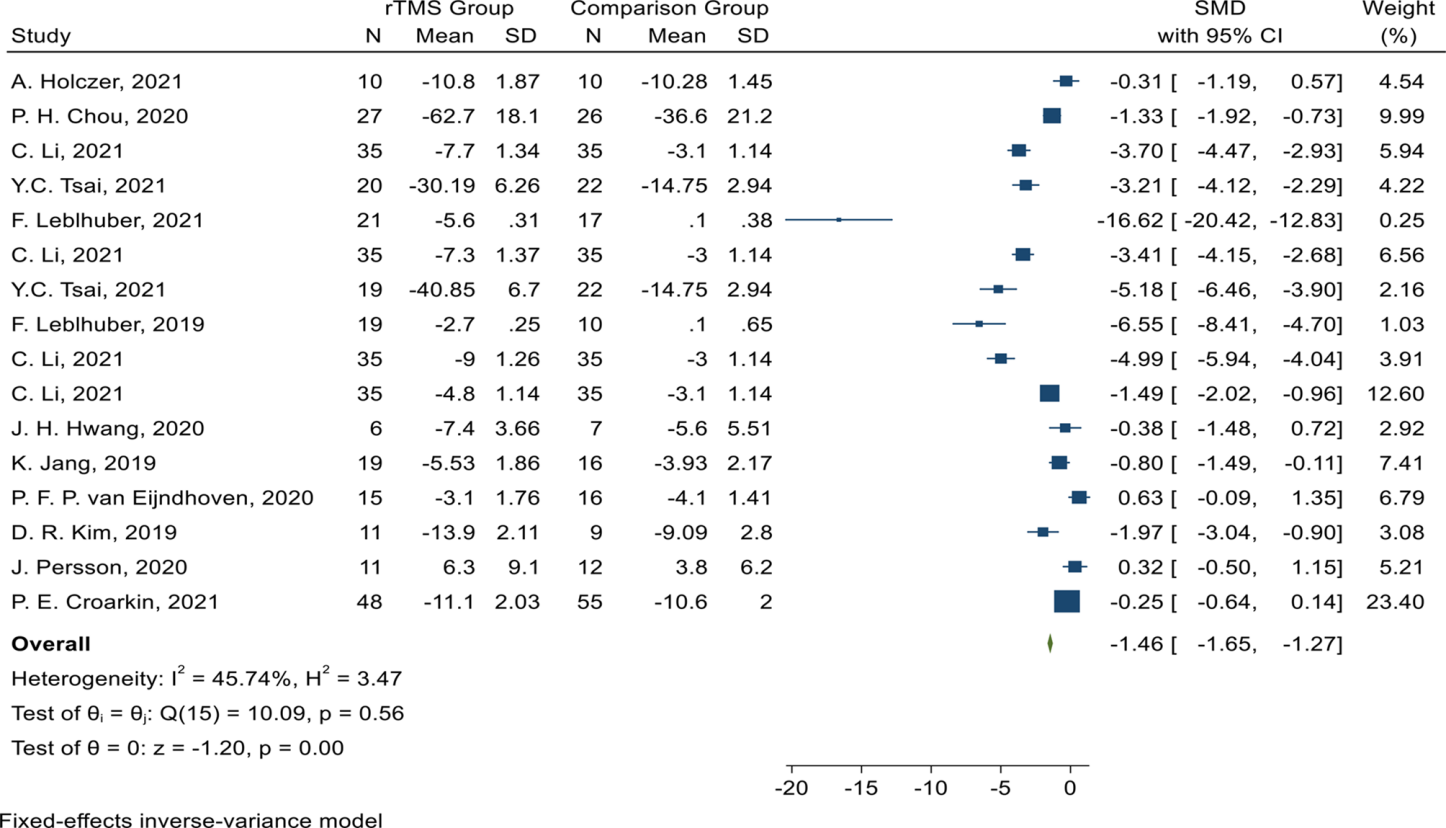
Forst plot of the effect of rTMS intervention on the Hamilton Depression Rating Scale (HDRS) in patients with major depressive disorder (MDD) without psychotic features evaluation scales (SMD: standardized mean differences, N: sample size, SD: standard deviation)

Out of the 24 selected studies, 16 effect sizes were extracted in terms of SMD and combined together. The largest and smallest effect sizes reported in these studies were from F. Leblhouber et al. and P. F. P Van Eijindhoven et al., respectively. After combining these studies, the meta-analysis results showed that the use of rTMS in patients with MDD without psychotic symptoms can reduce the mean HDRS score by 1.46 (SMD: -1.46; % 95 CI: -1.65, -1.27) (Fig. 2). The results indicated that the level of heterogeneity was significantly low in this analysis, indicating high homogeneity of the combined studies (I _square_: 45.74%; P _heterogeneity_: 0.56) (Fig. 2).

**Fig. 3:**
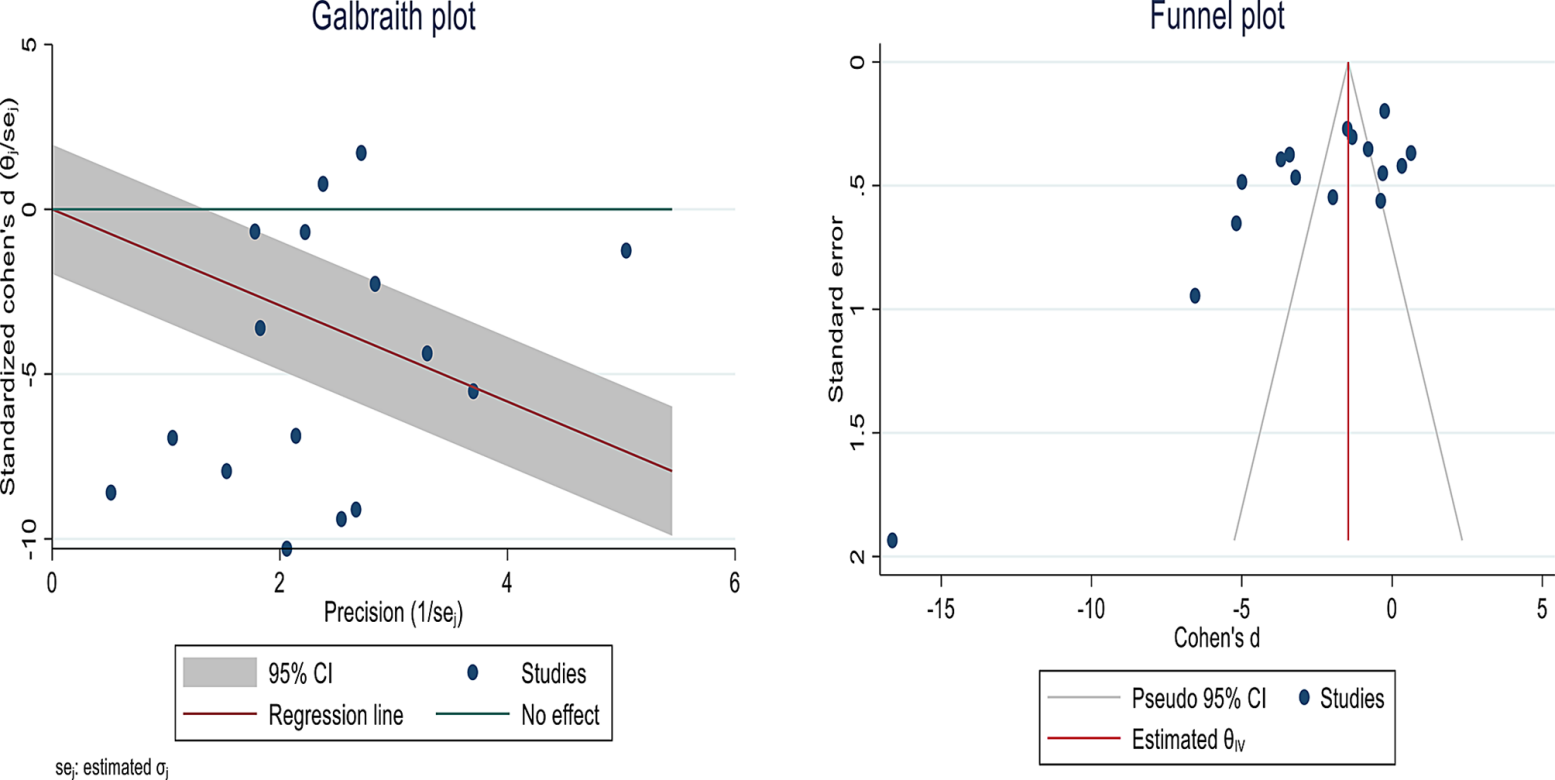
Galbraith and funnel plot of the effect of rTMS intervention on the Hamilton Depression Rating Scale (HDRS) in patients with major depressive disorder (MDD) without psychotic features evaluation scales

Heterogeneity and publication bias analyses were examined and reported using Galbraith and Funnel plots (Fig. 3). The results showed that publication bias was associated with the use of rTMS on mean HDRS scores in patients with MDD without psychotic symptoms (B: -9.21; SE: 1.445; P value: 0.0001). A trim and fill analysis was also performed due to the significant publication bias, but the results showed that this bias did not have a significant impact on the overall results (Fig. 3).

Subgroup analyses were conducted to determine the effect of rTMS on mean HDRS scores in patients with MDD without psychotic symptoms based on the number of treatment sessions, device power, and study population. The results are presented in Table 3. The results showed that the SMD in HDRS scores varied significantly depending on the number of treatment sessions, with a decrease of 2.60 units (SMD: -2.60; % 95 CI: -2.86, -2.33, I _square_: 55.12%; P _heterogeneity_: 0.55) for patients receiving 10 or fewer treatment sessions, a decrease of 0.28 units (SMD: -0.28; % 95 CI: -0.65, -0.09, I _square_: 39.91%; P _heterogeneity_: 0.89) for patients receiving 11 to 20 treatment sessions, and a decrease of 0.25 units (SMD: -0.25; % 95 CI: -0.64, -0.01, I _square_: 55.80%; P _heterogeneity_: 0.08) for patients receiving more than 21 treatment sessions.

**Table 3:** The effect of rTMS intervention on the Hamilton Depression Rating Scale (HDRS) in patients with major depressive disorder (MDD) without psychotic features evaluation scales based on Sessions, Power, and study population

Variables	Categories		SMD (% 95 CI)	Heterogeneity assessment		
				I Square	*P* value	Q test
HDRS	Sessions	< 10 Session	-2.60 (-2.86, -2.33)	55.12%	0.55	8.60
		11–20 Session	-0.28 (-0.65, -0.09)	39.91%	0.89	9.99
		> 21 Session	-0.25 (-0.64, -0.01)	55.80%	0.08	19.91
	Power	1 HZ	-3.12 (-4.05, -2.19)	44.32%	0.60	5.79
		10 HZ	-0.49 (-0.77, -0.21)	72.20%	0.04	11.36
		> 10 HZ	-2.97 (-3.31, -2.62)	66.09%	0.04	22.10
		Not Reported	-1.10 (-1.51, -0.70)	55.80%	0.09	12.91
	Study Population	MDD	-2.02 (-2.28, -1.77)	63.34%	0.05	15.11
		TDR	-0.98 (-1.29, -0.68)	29.00%	0.32	1.00
		Others	0.07 (-0.59, 0.73)	37.46%	0.43	7.17

HZ: Hertz; MDD: major depressive disorder; TRD: treatment resistant depression, SMD: Standardized Mean Differences, CI: Confidence Interval, Q: Q Cochrane Test

Based on the frequency of the rTMS device, the mean HDRS score decreased by 3.12 units (SMD: -3.12; % 95 CI: -4.05, -2.19, I _square_: 44.32%; P _heterogeneity_: 0.60), 0.49 units (SMD: -0.49; % 95 CI: -0.77, -0.21, I _square_: 72.20%; P _heterogeneity_: 0.04), and 2.97 units (SMD: -2.97; % 95 CI: -3.31, -2.62, I _square_: 66.09%; P _heterogeneity_: 0.04) in patients with MDD without psychotic symptoms when the device frequency was 1 Hz, 10 Hz, and over 10 Hz, respectively (Table 3).

Based on the overall analysis, the results showed that in patients with MDD, rTMS reduces the mean HDRS score more than other patients such as those with TDR, etc. (SMD: -2.02; % 95 CI: -2.28, -1.77, I _square_: 63.34%; P _heterogeneity_: 0.05) (Table 3).

**Fig. 4:**
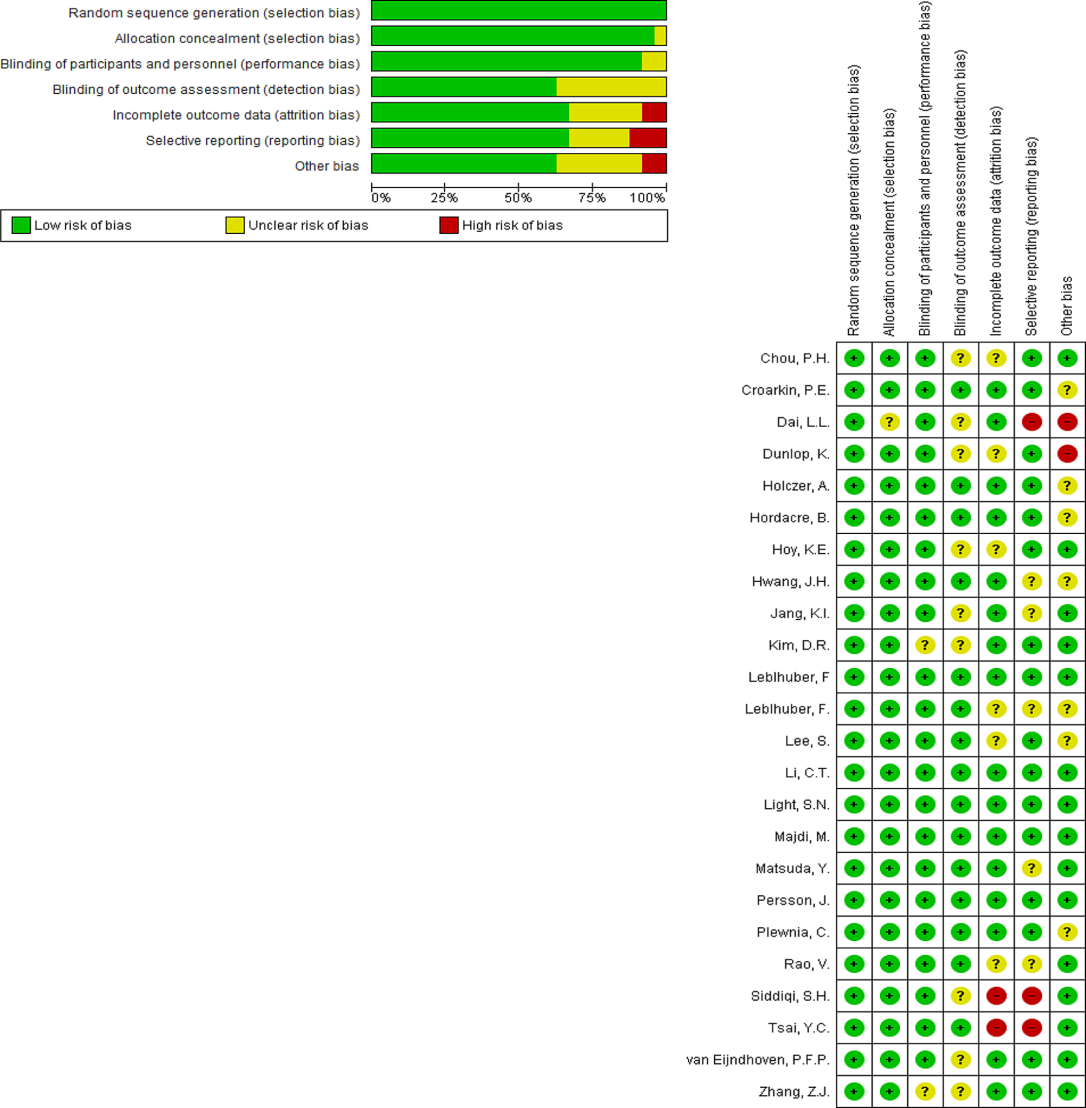
Risk of bias graph summary (review authors’ judgement about each risk of bias item presented as percentages across all included studies)

Figure 4 presents a summary graph of the risk of bias assessment, indicating the number of studies judged to be at low, unclear, or high risk of bias in each domain or overall (Fig. 4). In the analysis of risk of bias using the Cochrane checklist for assessing intervention studies, the results indicated that most of the studies included in the current meta-analysis were of adequate quality and were categorized as low risk of bias in terms of bias occurrence. A small number of studies were placed in the high risk of bias category for incomplete outcome data, selective reporting, and other bias, but they constituted a small percentage of the selected studies. Regarding the examination of individual clinical trial studies, the studies by Dal, L. L., Dunlop, K., Siddigi, S. H., and Tsal, Y. C. were biased in terms of incomplete outcome data and selective reporting (Fig. 4).

## Discussion

The results of this study, as demonstrated by the meta-analysis, indicate that the use of rTMS in patients with MDD without psychotic symptoms significantly reduces the mean HDRS score. The low level of heterogeneity observed in this analysis suggests high homogeneity among the combined studies and a definitive effect of rTMS. This study suggests that rTMS could be considered an effective therapeutic method for patients with primary depression without psychotic symptoms, as meta-analysis can provide more precise results. However, the mechanism of rTMS effect is still unknown. One hypothesis is that stimulation of specific areas of the brain cortex alters pathological activity in a network of gray matter regions involved in regulating mood. Additionally, rTMS may act through neuroplasticity, leading to increased expression of neurotrophic factors derived from the brain and structural changes, such as increased hippocampal volume. Further research, including randomized controlled trials and long-term investigations of the effects of rTMS on patients, is needed to better understand the mechanism of rTMS and to provide more accurate results [51–54].

A large number of studies have been conducted on the use of rTMS in the treatment of major depression, but they have shown different results. Some of these studies have shown similar results to the present meta-analysis, while others have reached different conclusions [55–61]. Generally, the results of studies indicate that rTMS can be an effective therapeutic method for patients with major depression, but this method is not effective for all patients and each patient needs to be evaluated separately. Additionally, the method of using rTMS and its various parameters are very important for optimizing the desired outcomes. In this study, different criteria such as the number of sessions, rTMS frequency and study population were considered to evaluate the effectiveness of rTMS on patients with MDD without psychotic symptoms. The analysis shows that with an increase in the number of rTMS sessions, the improvement in the HDRS score decreases, indicating a decrease in the patient’s response to rTMS with an increase in the number of sessions. Additionally, the effect of rTMS varies at different frequencies, which may be due to errors in measuring the outcome or the method of analysis chosen for this study. The next category is the study population, which has shown that rTMS has the greatest effect on patients with MDD compared to patients with TRD and other patients.

rTMS is a non-invasive method that uses a magnetic field to affect specific regions of the brains of individuals with MDD. This method can influence the activity of brain neurons and thereby help alleviate depressive symptoms [62–64]. In patients with MDD, the activity of neurons in specific regions of the brain that are involved in emotional regulation is altered. By applying the magnetic field of rTMS to these regions and altering the activity of neurons, it can help reduce symptoms [65, 66]. Some studies have shown that rTMS can lead to an increase in the levels of neurotransmitters such as serotonin and dopamine, which play a role in improving emotional state and depression [67–70].

It should be noted that the exact mechanism by which rTMS affects neuronal activity is not yet fully understood, and due to the high complexity of brain function, different methods of investigating the effects of rTMS on depression are available, each of which yields different results. However, rTMS is recognized as an effective therapeutic method for some patients with depression. It appears that the effect of rTMS varies depending on the frequency used. It is believed that high-frequency stimulation of the superficial cortex stimulates the target neurons and is usually used to activate the left prefrontal cortex. In contrast, low-frequency stimulation of the superficial cortex inhibits brain cortical activity and is usually directed towards the right prefrontal cortex [71].

The previous meta-analysis conducted by M. T. Berlim et al. in 2013 had included fewer clinical trial studies to determine the effect of rTMS on patients with MDD compared to the current meta-analysis. Additionally, subgroup analyses based on important and influential patient variables were not performed in the meta-analysis by M. T. Berlim et al. [72] due to the insufficient number of studies. On the other hand, in the current meta-analysis, subgroup analyses based on these variables were conducted. The results of the meta-analysis published in 2013 showed that the use of rTMS on patients with MDD may be effective, which is consistent with the results of the current meta-analysis [72]. However, the current meta-analysis specifically considered the change in the mean HDRS score as the outcome. In the overall analysis, the results of the current meta-analysis showed that the mean change in this outcome for patients with MDD was a reduction of 1.46 units on average, indicating an improvement in this measure in patients. The strengths of the current meta-analysis include screening of a large number of articles and considering a specific outcome and performing subgroup analyses based on important and influential variables. One of the major limitations of the current meta-analysis is the small number of intervention studies on the effects of rTMS on other outcomes or other assessment criteria for major depressive disorder (MDD), which should be considered in future studies.

## Conclusion

The use of rTMS has a significant and acceptable effect on mean HDRS in patients with MDD, according to the results of the current meta-analysis. In view of the inconsistent results of previous studies, this study has the potential to have a significant positive impact on the updating of treatment and care guidelines and on clinical decision-making. These findings suggest that treatment parameters like session frequency and power level should be considered to determine rTMS effectiveness in MDD. Our analysis identifies the optimal category (< 10 sessions) and power (1 Hz) for rTMS treatment design.

## Electronic supplementary material

Below is the link to the electronic supplementary material.

Supplementary Material 1

## Data Availability

The datasets during and/or analyzed during the current study are available from the corresponding author on reasonable request.
